# Effect of a lifestyle-integrated functional exercise (LiFE) group intervention (sLiFE) to falls prevention in non-institutionalized older adults. Protocol of a randomised clinical trial

**DOI:** 10.3389/fpubh.2023.1304982

**Published:** 2024-01-08

**Authors:** Inés Llamas-Ramos, Rocío Llamas-Ramos, Cristina Lugones-Sánchez, Susana González-García, Olaya Tamayo-Morales, Jorge Juan Alvarado-Omenat, Carmen Pablos-Hernández, Manuel A. Gómez-Marcos, Luis García-Ortiz, Emiliano Rodríguez-Sánchez

**Affiliations:** ^1^Institute of Biomedical Research of Salamanca (IBSAL), Primary Care Research Unit of Salamanca (APISAL), Salamanca, Spain; ^2^Department of Nursing and Physiotherapy, Universidad de Salamanca, Salamanca, Spain; ^3^Health Service of Castilla and Leon (SACyL), Salamanca, Spain; ^4^University Hospital of Salamanca, Salamanca, Spain; ^5^FisioSport Salamanca, Salamanca, Spain; ^6^Department of Medicine, Universidad de Salamanca, Salamanca, Spain; ^7^Department of Biomedical and Diagnostic Sciences, Universidad de Salamanca, Salamanca, Spain

**Keywords:** physical activity, prevention, falls, older adults, balance

## Abstract

**Introduction:**

Personalized programs of integrated strength and balance activities have been shown their effectiveness in falls reduction in the older adults.

**Objective:**

To measure whether a group intervention with the strength and balance principles of the sLiFE program is more effective than standard health advice in reducing the incidence of falls.

**Methods:**

The study will comprise 650 participants with more than 65 years who live at home, observing established inclusion and exclusion criteria. Participants will be randomly assigned in two groups: group intervention (*n* = 325) and standard health advice (*n* = 325). The intervention group will follow the balance and strength activities described in the LiFE program manual. The group intervention will be carried out in groups of 12–14 and will consist of seven one-hour sessions over 12 weeks in health centres. Incidence of falls and quality of life will be assessed as primary outcome variables. Fear of falling and exercise adherence will be analysed as secondary outcome variables.

**Discussion:**

Physical activity has been put forward as an effective treatment technique for these patients; however, long-term adherence to these programs remains a challenge. Group interventions could reduce dropout rates.

**Conclusion:**

Falls represent a major health problem globally due to the disability they cause in older people. Prevention would help reduce not only their incidence but also the health costs derived from their treatment. Group intervention helps clinicians to save resources and time, being able to attend more people with the same quality of care.

**Clinical trial registration:**

https://clinicaltrials.gov/study/NCT05912088?distance=50&term=NCT05912088&rank=1, identifier NCT05912088.

## 1 Introduction

Practitioners and researchers are focused on ageing and frailty, as demonstrated by different initiatives (1, 2). It has been shown that in the population older than 70 years, frailty represent a 5.5 times higher adjusted risk of mortality, a 2.5 times higher risk of new disability, and a 2.7 times higher risk of loss of mobility (3). To reduce frailty, action must be taken on its main risk factor, sedentary lifestyle (4), and the prevention of falls is thus of particular importance; falls are among the five main health problems related with disability in people older than 60 years (5).

Approximately 30% of people aged over 65% and 50% of those over 80 who live in the community have one fall once a year as a minimum (3, 5, 6). Similarly, about 30% of those who fall suffer a new fall in the same year and 10% suffer several falls (7). Falling is, therefore, a risk factor for further falls. Furthermore, Noureldin et al. found that 15% of hospitalised older adults people had a fall within 30 days of discharge (8).

Falls represent a major cause of disability in older adults and over 50% present sequelae (5, 6); half of those suffering a fracture from a fall do not fully recover their previous functional level. Older adults are admitted to hospital for injuries related to falls more frequently and they also present discharges and consecutive readmissions over the following three years (8, 9). Besides, between 32% and 80% of patients who survive hospitalisation after a hip fracture are left with a permanent disability (10), being a 95% of hip fracture cases a result of falls (11). In this regard, it has been observed that physical exercise approaches can reverse the functional disability caused by hospitalisation in older patients (12).

Several studies have demonstrated improvements at cardiovascular and mental levels (dementia), and in psychological stress and quality of life (13, 14). Exercise programs, multifactorial strategies for fall prevention and home interventions diminish falls (6, 15). Other systematic reviews and meta-analyses which have found multifactorial interventions, including exercise, postulated as the most effective, with a single intervention with exercise also showing significant effects are in this line. The literature suggests that exercise is the best approach for preventing falls at this population, but this could be influence by the exercise component selected (16, 17). Other structured training programs aim to improve muscle strength and balance, for example, the Otago Exercise Program (18). Nevertheless, they often do not generate long-term change, participation and adherence (19, 20); however, physical activity has shown numerous benefits (21). A review concluded that multicomponent group exercise and exercise at home, as well as safety interventions at home, diminish the rate and the risk of falls (22).

The LiFE study (Lifestyle-integrated Functional Exercise) intervention stood out as achieving the best results in preventing falls with a reduction of 13% (23). It is a personalized program which has demonstrated its effectiveness improving balance, strength, and physical activity, while falls in older adults were reduced by incorporating exercise activities into their daily activities. This program shown a clinically significant reduction of 31% in fall rate in comparison to the control program. A 30% in falls reduction is like most interventions currently recommended for preventing falls in clinical guidelines. It has recently been suggested in a pilot study that LiFE could be effective administered in a group setting in comparison to an individual intervention (24).

This project aims to compare the standard health advice to the original LiFE program implemented in a group (sLiFE), with the aim of facilitating large-scale implementation with lower use of resources and verifying effectiveness in terms of fall rates, physical activity, and profitability.

The objectives will be to assess if a group intervention implementing the sLiFE program principles reduces fall rates compared to standard advice; to assess whether fall prevention is more efficient in the group intervention than standard individual recommendations; to measure the incidence rate of falls according to participants’ level of physical activity; to assess medium- and long-term adherence to the exercise program and to find out the participants’ fear of falling.

## 2 Hypothesis

The sLiFE group intervention is more effective than usual health advice for the prevention of falls in older adults people living at home.

## 3 Methods

### 3.1 Design

Multicentre randomised clinical trial with two parallel arms, designed according to the CONSORT statement. This protocol has followed the SPIRIT guidelines for randomised trials. It was registered with ClinicalTrial.gov in June 2023 under the identifier NCT05912088.

### 3.2 Sample/participants

The study population will comprise subjects aged over 65 years who be in agreement to take part in the study and are being treated in the primary care setting of the Health Area of “X.” This study was reviewed and approved by the Salamanca Drug Research Ethics Committee in July 2022 under registration number PI 2022 071126. Before the study onset, all participants will be informed of the study objectives and will sign an informed consent form. Throughout the study the standards established in the Declaration of Helsinki will be followed. All those meeting the inclusion criteria in the health centres will be invited to participate.

#### 3.2.1 Inclusion criteria

older adults older 65 years, living at home and speaking and reading Spanish.

#### 3.2.2 Exclusion criteria

Heart failure (NYHA class III-IV); previous stroke (<6 months); Parkinson’s disease diagnosis; in active cancer treatment (last 6 months); chronic obstructive pulmonary disease (GOLD class III-IV); lower extremity fragile fracture; lower extremity amputation, treatment for depression less than six months ago, resting systolic pressure blood pressure > 160 or diastolic pressure > 100 uncontrolled; unavailable for the intervention, having more than two months travel or transfers planned in the first six months of the study; cognitive impairment moderate–severe (Mini Mental Cognitive Assessment <23); simultaneously participation in another clinical intervention trial.

### 3.3 Sample size

The main variable of the study was used to estimate the sample size, the annual incidence rate of falls in this population. An alpha risk of 0.05 and a beta risk of 0.2 (Power for Chi square 80% and t-test 94%), effect size estimated 0.30 (Cohen D) and 0.01 (V de Cramer) in a two-sided contrast has been accepted, 325 participants in the intervention group and 325 in the control are needed to find differences of ten percentage points as statistically significant between the control group, expected to be 30% [the estimate of falls in people older than 65 years of age (3, 5, 6)] and the intervention group, expected to be 20%. A give up rate of 10% during follow-up has been estimated.

This data has been calculated following the formula for qualitative variables published by Argimon Pallàs et al. (25).

$N=\frac{Za.Ö\;2.p.\left(1-p\right)+ZbÖ\;p1.\left(1-p1\right)+p2.\left(1-p2\right)2}{\left(p1-p2\right)2}$

### 3.4 Participant assessment

An external researcher will receive the participants and carry out the initial evaluation. The interview will be completed with information from the participants’ primary care medical history and the records of the University Hospital of “X.” The visit will take around 50–60 min and includes the collection of sociodemographic data, lifestyle, physical activity, cognitive status, adherence to exercise, quality of life and fear of falling. Participants will be fitted with a digital pedometer to record their physical activity for eight days. After the initial assessment, participants will be randomized to the intervention or control group.

### 3.5 Randomisation

**Figure 1:**
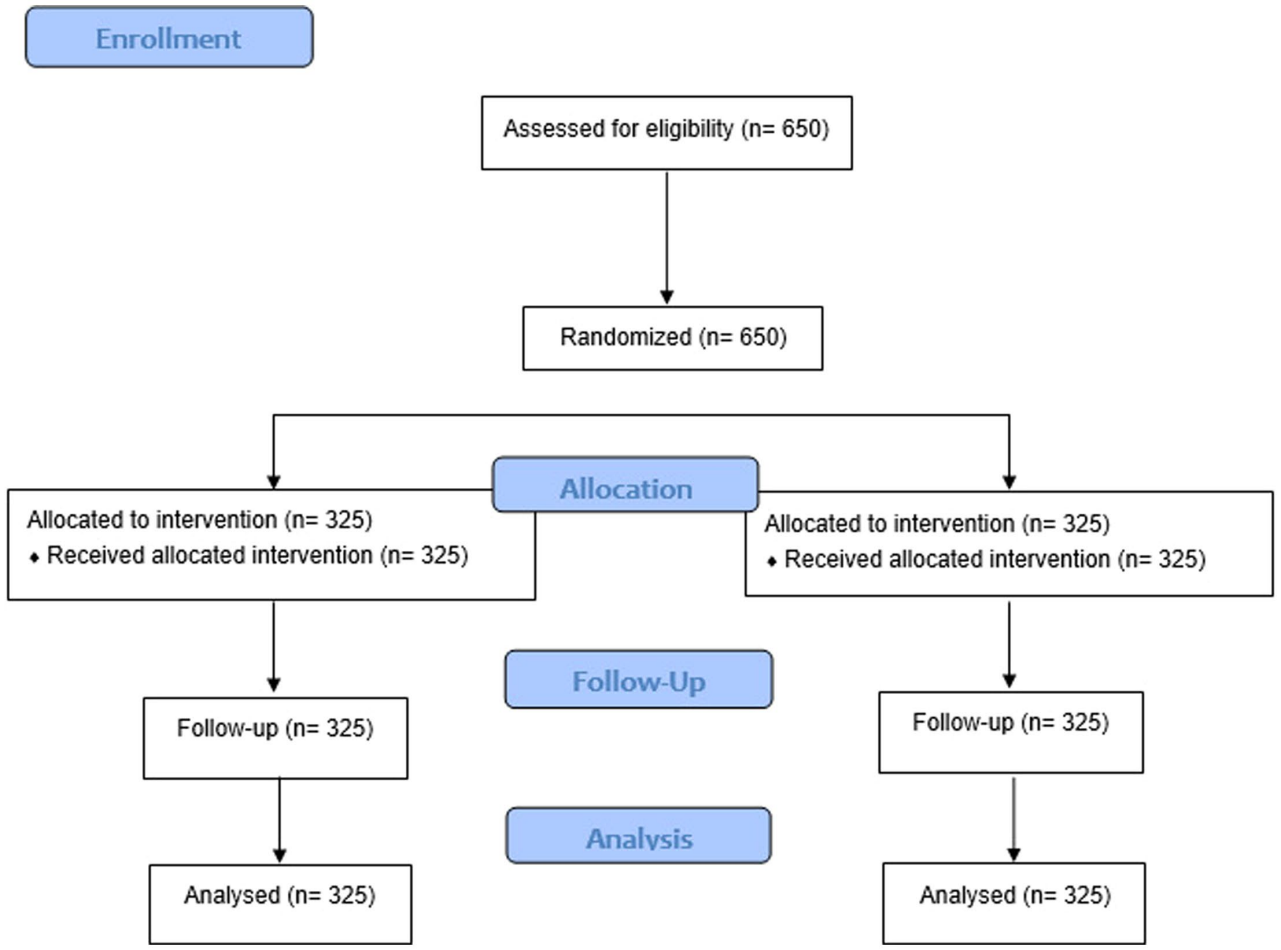
Sample size flow chart.

Once the inclusion criteria have been assessed, participants will sign informed consent and will then be randomised into the intervention/control group (Figure 1). An independent investigator, blinded until groups have been assigned will be in charge of generate the allocation sequence generated in a 1:1 ratio using the Epidat 4.2 software package. Considering the nature of the study, participants cannot be blinded to the intervention.

### 3.6 Procedure for the sLiFE program intervention

The intervention will take place in four different stages.

#### 3.6.1 Stage one

Physiotherapists (who led the sessions) will establish the guidelines to be followed and the intervention dynamics (sessions will be carried out in the same way and with the same contents in all health centres). The manuals for professionals, in Spanish, will be used to guarantee the reproducibility (26).

#### 3.6.2 Stage two

The intervention group (12–14 participants) will be given a brief guide in Spanish from the participant’s manual to advise them in carrying out the activities. The intervention group (*n* = 325) will do the balance activities (tandem stance, tandem walk, one-legged stand, leaning from side to side, leaning forward and backward, stepping over obstacles forwards and backwards, stepping over obstacles sideways) and strength activities (knee bends, sitting down and getting up from normal and low chairs, toe stand, toe walk, heel stand, heel walk, walking sideways, climbing stairs, and tightening muscles), the principles and implementation strategies (26). A total of five one-hour in-person sessions and two follow-up telephone sessions will be held.

#### 3.6.3 Stage three

Implementation of the sLiFE program (26). The total intervention comprises twelve weeks according to the following schedule (Table 1).

**Table 1:** Session schedule.

Session/week	Balance	Strength
1 (on-site)/Week 1	*Tandem stance *Tandem walk	*Knee bends *Sitting down and getting up (normal chair/low chair)
2 (on-site)/Week 2	*Standing on one leg	*Toe stand *Toe walk *Heel stand *Heel walk
3 (on-site)/Week 3	*Leaning side-to-side *Leaning forwards and backwards	*Walking sideways *Climbing stairs
4 (on-site)/Week 4	*Stepping over obstacles forwards and backwards *Stepping over obstacles sideways	*Flexing muscles
5 (on-site)/Week 6	Rest	Rest
6 (telephone)/Week 8	Follow-up	Follow-up
7 (telephone)/Week 12	Follow-up	Follow-up

#### 3.6.4 Stage four

Follow-up evaluation at six months.

Spirit figure has been added as Supplementary material.

### 3.7 Variables

#### 3.7.1 Sociodemographic variables

Participants’ age, education level, marital status, and profession will be noted. The prescribed pharmacological treatment, lifestyle habits, smoking history and smoking pattern and alcohol consumption will be collected.

#### 3.7.2 Anthropometric variables

Height will be measured with the portable Seca 222 system, with the subject standing. The average of two measurements, rounded to the nearest centimetre, will be recorded. Weight will be measured using a SOEHNLE 7830 digital column scale.

Waist and hip circumference will be measured twice, the recommendations of the Spanish Society for the Study of Obesity will be followed.

Systolic and diastolic blood pressure will be measured with a validated OMRON M10-IT blood pressure monitor (Omron Health Care, Kyoto, Japan), the recommendations of the European Society of Hypertension will be followed.

#### 3.7.3 Charlson Comorbidity Index

The Charlson Comorbidity Index (CCI) was developed in 1987 by Mary E. Charlson. It has been considered the gold-standard tool in clinical research as a prognostic index to predict mortality. This index is a standardized score calculated as a simple weighted sum of comorbidity item scores (27, 28). The original version of the CCI was composed on 19 items which correspond to different clinical comorbidities (27).

#### 3.7.4 Frailty

Frailty will be measured following the five criteria of Fried’s phenotype (29): (1) Low muscle strength; (2) Poor nutrition; (3) Poor endurance; (4) Slow walking; and (5) Low physical activity. Participants who meet these criteria will nevertheless be classified as active if they reported a high amount of daily usual physical activity (climbing stairs or lifting weights). The outcome will be meeting one frailty criterion at least.

#### 3.7.5 Physical activity

Physical activity will be assessed with a digital pedometer (Omron HJ-321 Tri-Axial) (30), to be placed front and middle on one thigh for a period of 8 consecutive days (30). In addition, the Global Physical Activity Questionnaire (GPAQ) will be used (31). This questionnaire is made up of 16 questions about PA carried out in a typical week, differentiating between the different types of activity in work, travel and free time. Data is collected on intensity (low/moderate/high), frequency (days / usual week) and duration (hours–minutes/typical day) of physical activities carried out in three domains: (1) work (paid employment or unpaid, study, housework or job search), (2) commuting (walking/cycling to get from one place to another), and (3) free time (leisure). A question is also included about sedentary behaviour (time usually spent sitting or lying down, excluding time spent sleeping at night).

#### 3.7.6 Mobility

The Short Physical Performance Battery (SPPB) assesses three aspects of mobility: balance, gait speed and strength of limbs or lower limbs to get up from a chair (32).

#### 3.7.7 Cognitive performance

The Montreal Cognitive Assessment (MOCA) (33) determines the existence of mild cognitive dysfunctions. It comprises 30 questions and takes 10–12 min to complete.

#### 3.7.8 Primary outcome measures

- The incidence rate of falls will be estimated based on the intervention and control group. Falls will be recorded using a daily log sent to the study centre monthly. On suffering a fall, participants must provide information about the time, date, injuries and prescribed treatment in relation to the fall, the fall location, and the movement which cause the fall. The person will be interviewed by telephone to correct any lack of data to determine the injuries details and to confirm their health status at this time (7).
- Quality of life will be measured through the EuroQol 5D questionnaire, validated in Spanish (34). This questionnaire comprises five items (mobility, personal care, daily activities, pain/discomfort, and anxiety/depression) and a self-assessed thermometer of health status.

#### 3.7.9 Secondary outcome measures

- Fear of falling: Short Falls International Scale of Efficacy (Short FES-I) assess “concerns about falling” (35). The scale is the falls efficacy scale-international short version, comprising seven items (items 2,4,6,7,9,15 and 16). Item responses are coded on a 4-point Likert scale: (1) not at all concerned, (2) somewhat concerned, (3) fairly concerned, and (4) very concerned.
- Exercise adherence will be measured using the Exercise Adherence Rating Scale (EARS) as part of the schedule sent in monthly. This scale is composed of 16 items, scored using a 5-point Likert scale (0 = completely agree to 4 = completely disagree) with a total summed score range from 0 to 64 (36).
- Cost-effectiveness of the intervention: Incremental cost-effectiveness ratio (ICER) related with the ratio of the difference in costs to the health effects difference in both interventions (37). These costs include outpatient treatment, formal/informal care, medication, transportation, room rental, intervention costs involving labour costs, staff and participants transportation, and materials.

### 3.8 Procedures

Six months after the initial control group assessment and six months after the intervention group sessions have finished, subjects of both groups will be assessed with the same tests that were carried out in the initial assessment. After random assignment, follow-up assessments will be performed by the blind group assignment assessors. The database used in this study will only display information unrelated to the intervention when they are logged in to ensure the blinding of assessors. Should a participant wish to withdraw from the study, they will continue to be eligible to complete the follow-up measurements with their consent. Researchers will record the reasons and date of withdrawal, but data recorded before withdrawal will be used unless the participant decide to use their right to have all data deleted.

Study data will be collected and managed using REDCap electronic data capture tools, hosted at the University of “X.” REDCap is a secure, web-based software platform designed to support data capture for research studies.

### 3.9 Statistical analysis

The study population baseline characteristics will be expressed as means ± standard deviation (SD) for quantitative variables and in frequency distributions for categorical variables. Student’s t-test, chi-square, and Fisher’s exact tests will be applied to find differences in baseline characteristics between intervention and control groups. All analyses of the variables obtained from the questionnaires will be analyzed using the reliability and validity criteria proposed by their authors.

The main analyses will be performed on the intention-to-treat principle, so all randomised subjects will be included in the data analysis set for which the initial assessment was performed. Participants who withdraw or drop out will be asked to be included in follow-up measurements; those lost to follow up will be considered in the full set of analyses as missing data. Detailed modelling of variations between participants and groups will be done in terms of factors such as dose, acceptability, and contextual factors. Using the Chi-square test, we will compare the proportions of subjects who have had a fall in both groups. The comparison of all outcomes between baseline, six and twelve months will be carried out using the two-way repeated measures ANOVA. Logistic regression analysis will be conducted to determine the influence of the different risk factors on falls. Statistics analysed will be performed with SPSS V.25.0 statistical package (SPSS Inc., Chicago, Illinois, United States). *p* values for cut-off values for significance were established at <0.05 (two-tailed). Results were interpreted according *[Cohen]*, who considers small size if Cohen D are between 0.2 ≤ and < 0.5; average effect size if differences are between 0.5 ≤ and < 0.8 and very high effect size if differences are ≥0.8 (38).

### 3.10 Trial status

Nowadays people have been contacted to invite them to participate. First groups will be collected until the end of the year.

## 4 Discussion

Falls prevention is seen as one of the most needed interventions in the population aged over 65. Both the NICE guide on fall prevention (39) and the British and American Geriatrics Societies (6) recommend annual screening of subjects older than 65 years for a falls history and the presence of disorders in gait and balance.

While some publications have found evidence of the efficacy of a multifactorial intervention in reducing falls in older adults and/or their consequences (40), some interventions developed in primary care in Spain have not been able to reduce the frequency of falls (7, 40). However, the 2018 Cochrane review showed that most of these multifactorial and multicomponent studies were of low quality, and high risk of bias, and that there may be little or no effect on other fall-related outcomes. Furthermore, structured programs have failed to induce long-term behaviour change towards more regular exercise, demonstrating poor adherence (41). New concepts and formats with large-scale implementation and long-term adherence to balance and strength in exercise are required urgently.

The intervention of the LiFE study has achieved the best results in preventing falls (23) and is considered to be of high quality. Moreover, the LiFE program in terms of function and participation, was superior providing support for this program in measuring both frailty and fall risk. However, less than 10% of older people regularly do strength training and probably even less do balance activities. In the LiFE program, adherence was significantly better (23) and exceeded the 42% adherence reported in the New Zealand Otago trial; this trial tested a successful structured and home-based exercise program (18). However, despite its effectiveness, the implementation of the home-based program LiFE requires considerable economic costs and human resources. Nevertheless, no study has yet been implemented comparing the LiFE intervention group format to standard health advice in a larger population.

A group intervention could facilitate adherence to these activities and clinicians could save resources and time to be more effective and to guarantee the care quality.

For these reasons, the sLiFE program aims to promote the performance of physical activity centered on modifying the behaviour of participants and has demonstrated its effectiveness in a large randomised controlled trial to risk of falls reduction. It seems necessary to assess whether the sLiFE program intervention implemented in a group format can be recommended over individual participation.

### 4.1 Limitations

The 24 months prior to data collection could be a long period to remember such events for such individuals. In this study the interview will be complemented with the information available in the participants’ primary care medical history and in the records of the University Hospital “X” to ensure that all falls which had consequences are considered.

## 5 Conclusion

This project can help to increase physical activity are effective in falls reduction and in avoiding the consequences derived from them. The key characteristic is the analysis of whether a more economical (group) intervention can be recommended if similar results are obtained in comparison to the individual intervention carried out: LiFE program which has been shown a fall reduction in older adults. It could be applied to larger groups, and it would be possible to recommend it to most older people as the best fall prevention strategy.

## Data availability statement

The raw data supporting the conclusions of this article will be made available by the authors, without undue reservation.

## Ethics statement

The studies involving humans were approved by Salamanca Drug Research Ethics Committee. The studies were conducted in accordance with the local legislation and institutional requirements. The participants provided their written informed consent to participate in this study.

## Author contributions

IL-R: Conceptualization, Funding acquisition, Investigation, Methodology, Project administration, Supervision, Writing – original draft, Writing – review & editing. RL-R: Conceptualization, Investigation, Methodology, Writing – original draft, Writing – review & editing. CL-S: Methodology, Writing – review & editing. SG-G: Methodology, Writing – review & editing. OT-M: Methodology, Writing – review & editing. JA-O: Methodology, Writing – review & editing. CP-H: Methodology, Writing – review & editing. MG-M: Methodology, Writing – review & editing. LG-O: Formal Analysis, Methodology, Writing – review & editing. ER-S: Conceptualization, Funding acquisition, Investigation, Methodology, Project administration, Writing – original draft, Writing – review & editing.
