# Supplementary material for: Effect of a lifestyle-integrated functional exercise (LiFE) group intervention (sLiFE) to falls prevention in non-institutionalized older adults. Protocol of a randomised clinical trial
Source: Front Public Health. 2024 Jan 8;11:1304982. doi: 10.3389/fpubh.2023.1304982 (PMC10801183; doi:10.3389/fpubh.2023.1304982)
Supplement: Supplementary file 1 [file Table_1.DOC]

Supplementary material. Spirit figure

|  | **STUDY PERIOD** | | | | | | | |
| --- | --- | --- | --- | --- | --- | --- | --- | --- |
|  | **Enrolment** | **Allocation** | **Post-allocation** | | | | | **Close-out** |
| **TIMEPOINT** | ***-t_1_*** | **0** | ***t_1_*** | ***t_2_*** | ***t_3_*** | ***t_4_*** | ***etc.*** | ***t_x_*** |
| **ENROLMENT:** |  |  |  |  |  |  |  |  |
| **Eligibility screen** | X |  |  |  |  |  |  |  |
| **Informed consent** | X |  |  |  |  |  |  |  |
| ***Initial assessment*** | X |  |  |  |  |  |  |  |
| **Allocation** |  | X |  |  |  |  |  |  |
| **INTERVENTIONS:** |  |  |  |  |  |  |  |  |
| ***sLiFE Program*** |  |  |  |  |  |  |  |  |
| ***Control group*** |  |  |  |  |  |  |  |  |
| **ASSESSMENTS:** |  |  |  |  |  |  |  |  |
| ***Sociodemographic, anthropometric variables*** | X |  |  |  |  |  |  |  |
| ***Incidence of falls, quality of life*** | X |  |  |  |  |  | . | X |
| ***Exercise adherence, EARS, ICER]*** | X |  |  |  |  |  |  | X |
